# Electrokinetic and Hemostatic Profiles of Nonwoven Cellulosic/Synthetic Fiber Blends with Unbleached Cotton

**DOI:** 10.3390/jfb5040273

**Published:** 2014-11-28

**Authors:** J. Vincent Edwards, Elena Graves, Alvin Bopp, Nicolette Prevost, Michael Santiago, Brian Condon

**Affiliations:** USDA-ARS, Southern Regional Research Center, 1100 Robert E. Lee Blvd., New Orleans, LA 70124, USA; E-Mails: elena.graves@ars.usda.gov (E.G.); abopp@suno.edu (A.B.); nicolette.prevost@ars.usda.gov (N.P.); michael.santiago@ars.usda.gov (M.S.); brian.condon@ars.usda.gov (B.C.)

**Keywords:** nonwoven cotton, hemostasis, thromboelastography, electrokinetic properties, accelerated clotting, contact angle

## Abstract

Greige cotton contains waxes and pectin on the outer surface of the fiber that are removed when bleached, but these components present potential wound dressing functionality. Cotton nonwovens blended with hydrophobic and hydrophilic fibers including viscose, polyester, and polypropylene were assessed for clotting activity with thromboelastography (TEG) and thrombin production. Clotting was evaluated based on TEG measurements: *R* (time to initiation of clot formation), *K* (time from end of *R* to a 20 mm clot), α (rate of clot formation according to the angle tangent to the curve as *K* is reached), and MA (clot strength). TEG values correlate to material surface polarity as measured with electrokinetic parameters (ζ_plateau_, Δζ and swell ratio). The material surface polarity (ζ_plateau_) varied from −22 to −61 mV. *K* values and thrombin concentrations were found to be inversely proportional to ζ_plateau_ with an increase in material hydrophobicity. An increase in the swell ratios of the materials correlated with decreased *K* values suggesting that clotting rates following fibrin formation increase with increasing material surface area due to swelling. Clot strength (MA) also increased with material hydrophobicity. Structure/function implications from the observed clotting physiology induced by the materials are discussed.

## 1. Introduction

### 1.1. Polysaccharide-Based Nonwovens with Hemostatic Activity

In recent years nonwoven medical dressings have increased in usage both for absorbent hemostatic and chronic wound applications [1,2]. Some nonwoven dressings are composite materials containing more than one fiber, component, or layer [3,4]. A variety of natural and synthetic fibers have been reported and are used to promote hemostasis including cotton, viscose, rayon/polyester, glass filament, chitosan, nylon, wool and alginate [5,6,7,8,9,10,11,12,13,14,15]. Topical hemostatic agents consisting of proteinaceous materials like wool [10], collagen and fibrin [16] have been reported to be very effective in promoting clotting. However, natural hemostatic fibers are also derived from polysaccharide [17] fibers made from, oxidized regenerated cellulose [9], carboxymethylcellulose [4,15], *N*-acetyl-glucosamine [18,19], and starch [20], and these are incorporated into materials that constitute structurally or process modified polysaccharides fibers. These polysaccharide fibers have also played an important role in understanding the relationship of structure to function in the design of hemostatic dressings, and they have been examined for their structure/function relationships in contact activation of blood coagulation [6,18,19]. 

### 1.2. Thromboelastography for Assessment of Nonwoven Fiber Blends

Thromboelastography (TEG) assesses the viscoelastic properties of whole blood under low shear conditions and provides information about global hemostatic function from the beginning of clot formation through clot retraction and fibrinolysis. It is viewed as an effective tool to assess biomaterials for properties that may induce contact activation coagulation as relates to the physiology of the clotting process, and with respect to clotting factors, platelets and whole blood or plasma constituents in clot formation. 

Fiber properties including composition, pH, charge, wettability, surface area and polarity are useful in understanding structure *versus* function relations measured with TEG, and in some cases predicting the *in vivo* hemostatic performance of a material. These are properties of material clotting function that can often best be understood by varying material composition as presented here. The application of TEG to biomaterials has been documented for a variety of biomaterial types having neutral, anticoagulant and procoagulant profiles [21].

### 1.3. Greige Cotton Nonwovens

Cotton fibers are complete plant cells that grow out from cotton seeds [22]. The fiber is composed mainly of cellulose molecules [23], which are found in the primary and secondary cell wall, mostly in small crystallites. The crystallites are stabilized by conventional O–H···O hydrogen bonds as well as the weaker van der Waals forces and C–H···O hydrogen bonds [24]. The morphology of the cotton fiber consists of an outer protective cuticle which contains hydrophobic lipids that are only a few percent by weight of the total fiber [22]. The primary cell wall below the cuticle also contains pectin (approximately one percent by weight of the fiber), cellulose and some proteinaceous material. Greige cotton is unbleached cotton that still retains the waxes and pectin in the outer parts of the fiber that are otherwise removed by the finishing processes of bleaching and scouring. The potential to use greige cotton in nonwoven absorbent products has received increased attention based on innovations in cotton cleaning and nonwovens processes that open and expose the hydrophilic cellulosic component of greige cotton fiber to water absorption [25,26,27]. Previously it was shown that nonwoven greige cotton when compared with nonwoven bleached cotton reduces the TEG-determined clotting time for both initial formation of fibrin and clot formation by fifty percent and increases the rate of clot formation while retaining approximately the same clot strength [28]. 

This study further addresses the hemostatic properties of greige cotton when blended with other fibers of varying hydrophilic/hydrophobic surface polarity [28]. Here we report the hemostatic properties of a range of materials based on thromboelastography, and thrombin production and demonstrate the relation of hemostatic activity to fiber surface properties, as measured from electrokinetic analysis, in the nonwoven blends

## 2. Results and Discussion

### 2.1. Composition and Surface Chemistry

The composition, pattern, and densities of the greige cotton material blends are outlined in Table 1. The materials consist of varying ratios of greige cotton, viscose, polyester, and polypropylene. In this regard the fabrics varied in fiber composition, structural pattern, and density. Three types of 100% hydroentangled nonwoven greige cotton were prepared with either an apertured or random pattern structure at densities of 45 and 35 g/m^2^ (samples I–III). Three fine apertured fabric blends of greige cotton and viscose at 45, 35 and 25 g/m^2^ were also tested; blended were a 75/25 (25 g/m^2^), and two 50/50 greige cotton/viscose (35 and 45 g/m^2^) appertured nonwovens. Some more hydrophobic materials were prepared including a 40/30/30 polyester/greige cotton/viscose at 35 g/m^2^ and a blend with greige cotton/polypropylene (50/50) at 25 g/m^2^. A rayon/polyester and viscose material that are used routinely in hemostatic nonwoven wound dressings (12) are included to contrast with the structure/function properties of the nonwovens.

**Table 1:** Description and densities of greige cotton nonwovens.

Sample ID	Sample description	Density (g/m^2^)
I	100% greige cotton (fine aperture)	35
II	100% greige cotton	45
III	100% greige cotton (no pattern)	45
IV	75% greige cotton/25% viscose	25
V	50% greige cotton/50% viscose	35
VI	50% greige cotton/50% viscose	45
VII	40% polyester/30% greige cotton/30% viscose	35
VIII	50% polypropylene/50% greige cotton	25

**Figure 1:**
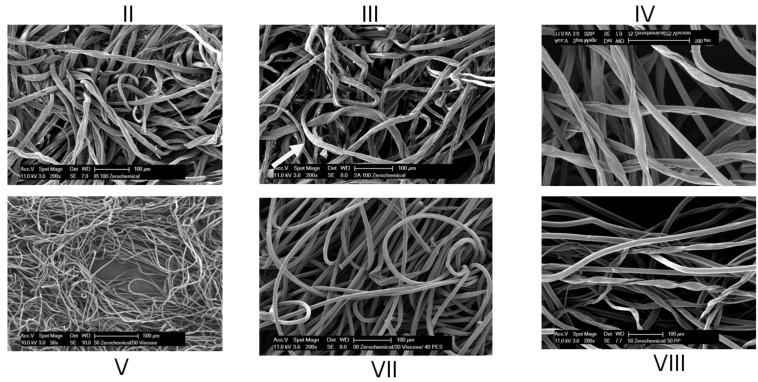
Scanning electron micrographs of select nonwoven as identified in Table 1 and described in the Experimental Section. Arrow in III points to cuticle loosening of outer fiber.

Scanning electron micrographs of select samples are shown in Figure 1. Evident in these images are the random interwoven structure resulting from hydroentanglement, and images of the greige cotton fibers reveal some fiber cuticle loosening from the primary cell wall as shown in the SEM of III (Figure 1).

The material surface polarity as determined by electrokinetic analysis are reported in Table 2, and was found to vary in a manner consistent with the blending of hydrobphoic and hydrophilic fibers. Zeta (ζ) potential decrease is caused by swelling of the fibers and outward movement of the aqueous shear plane ions that are in contact with the outer Helmholtz plane on the fiber surface [29]. Zeta potential decrease was observed for all of the materials of this study. Zeta potential titrations under these conditions give rise to a determination of the relative material polarity (ζ_plateau_) and are used here to assign the material surface polarity. The ζ_plateau_ varied from −61 to −18. In this regard zeta potential is a measure of the charge and charge density on the surface of the material and can be used to gauge the stability of a colloidal or the ability of a material to absorb liquid.

The one hundred percent polypropylene sample (VIII) had the most negative zeta potential (61 mV), and thus is the most hydrophobic. Whereas, the 50/50 viscose/greige cotton (sample VI) was the most hydrophilic (−18 mV) material. Thus, an increase in swelling of the material occurs with zeta potential decrease for all of the materials of this study. As the shear plane moves out to a more diffuse layer of ions a decrease in ζ-potential is observed [30,31]. This is evident in the samples of this study as seen in Table 2 where blending of the more hydrophobic synthetic polyester and polypropylene fibers with greige cotton gives a decrease in the ζ-potential and an increase in swelling. Similarly, in a converse mechanism to the one just described, the blends containing viscose, which is a more hydrophilic fiber, tend to make the ζ-potential less negative.

**Table 2:** Electrokinetic parameters of nonwovens as outlined in the Experimental Section.

Sample ID	Density (g/m^2^)	Moisture content (%)	IEP pH	Plateau potential (mV)	Swell test *K* (min^−1^)	ζ_0_ (mV)	ζ_∞_ (mV)	∆ζ	Swell ratio	*R*^2^
I	35	9.23	2.0	−27	0.018	−32.7	−29.4	0.101	1.054	0.939
II	45	7.34	2.2	−25	0.001	−25.9	−16.4	0.125	1.069	0.970
III	45	7.18	2.2	−26	0.008	−27.1	−23.5	0.132	1.073	0.953
IV	25	9.25	1.8	−25	0.006	−24.7	−22.6	0.083	1.044	0.973
V	35	9.68	2.1	−22	0.005	−21.1	−19.9	0.056	1.029	0.920
VI	55	9.86	2.2	−18	0.007	−18.9	−17.3	0.086	1.046	0.972
VII	35	6.86	2.1	−37	0.004	−35.5	−27.1	0.125	1.069	0.991
VIII	25	4.04	2.2	−61	0.011	−54.6	−45.3	0.171	1.098	0.961
Rayon-polyester	–	–	2.8	−16	0.030	22.3	16.6	0.255	1.158	0.991
Polypropylene	–	–	2.5	−32	0.032	66.1	41.8	0.370	1.259	0.989

**Figure 2:**
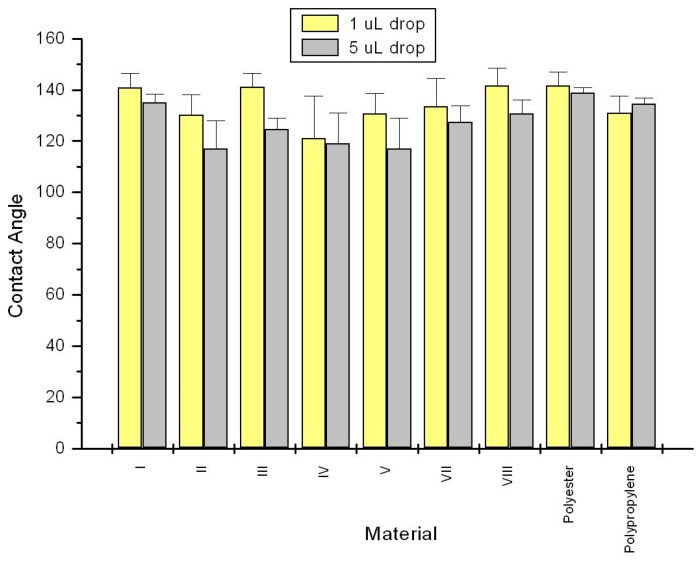
Contact angle results for nonwoven samples of this study as described in the Experimental Section. Description of the material composition is in Table 1.

As seen in Figure 2 contact angle measurements of the samples are consistent with the ζ_plateau_ values and reveal that all of the materials are relatively hydrophobic since all contact angles measured were greater than 90°. There is an exception to this with VI, which was too absorbent to give a reproducible contact angle. This is probably due to the density of the more absorbent viscose fibers as opposed to V which has a lower density. It is important to note that fabric surfaces are not smooth and wetting has been looked at by two simplified models that take into account rough surfaces: Wenzel [32] and Cassie [33]. In the Wenzel model [32], the liquid wets the entire surface while in the Cassie model [33] the liquid contacts only the tops of the surface irregularities. Either model explains higher contact angles for non-wetting rough surfaces compared to non-wetting smooth surfaces. The surface area of a rough surface will be greater than the surface area for a smooth surface given the same footprint. This would explain in part the relatively high contact angles for the fabric blends of this study.

### 2.2. Clotting Profiles

**Figure 3:**
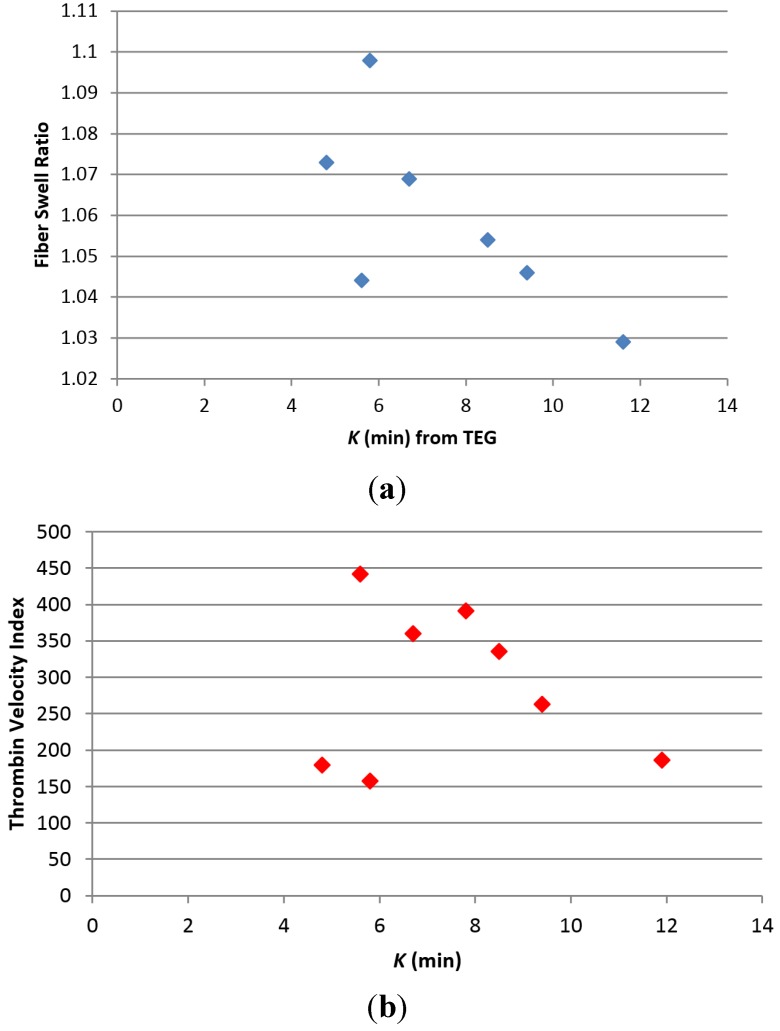
(**a**) Plot of fiber swell ratio (see Table 2) *versus* *K* (Table 3), the clotting time from fibrin formation to formation of a 40 mm clot; (**b**) Plot of Thrombin Velocity Index *versus* *K* (clotting time).

The results of the thromboelastography (TEG) profiles for all samples including greige cotton nonwoven blends are reported in Table 3. The three 100% greige cotton samples which varied in fabric pattern and density demonstrated some differences in the *R* and *K* values. For example the *R* value, which is the time to initial fibrin formation, was similar, for I and II (6.2 and 6.6 min. respectively) but was increased for III (9.9 min). However, the *K* value or time to formation of a 20 mm clot observed for III was much lower (4.8 min.) than that found for I and II, and was the shortest *K* time of any of the materials tested. It is interesting that an increase in swell ratio observed for the three completely greige cotton samples, corresponded to a decrease in *K* time. This relationship also exits for most of the nonwoven blends of this study as is shown in Figure 3a. A corresponding relationship of decrease in *K* to increase in the thrombin velocity index is shown in Figure 3b, and demonstrates that thrombin generation associated with the samples (Table 4), and determined independently of TEG analysis are consistent with the trend in *K* values.

The most pronounced effect on clotting times and rate of clotting of the nonwoven blends tested was observed in sample VIII which is a 50/50 blend of greige cotton and polypropylene. The TEG-determined time to fibrin formation (*R*) for VIII was 5.7 ± 1.0 min. and the clotting time (*K*) from fibrin formation to formation of a 20 mm clot was 5.8 ± 0.7 min, which were both approximately half those observed for blood alone. The rate of clotting (α) for sample VIII was the highest of any sample tested suggesting that the hydrophobic nature of these samples increases the rate of clotting.

**Table 3:** Results of the thromboelastography analysis. Values are described in the text *R*—time to fibrin formation; *K*—time from fibrin formation to clot formation (35 mm); angle—rate of clot formation; MA—strength of clot formation.

Sample	*R* (min)	Std dev *	*K* (min)	Std dev *	Angle (°)	Std dev *	MA (Mm)	Std dev *
Bovine Blood	11.6	0.6	7.0	1.3	35.4	0.2	25.0	2.1
Kaolin Control	4.2	0.1	4.9	0.1	54.6	4.2	26.0	0.5
Combat Gauze	5.5	0.8	5.3	0.4	25.6	1.1	31.1	1.1
Rayon/Polyester	4.9	0.3	7.8	1.5	24.6	2.6	31.6	0.1
I	6.2	0.9	8.5	0.1	19.5	0.9	27.0	0.5
II	6.6	0.2	7.8	0.6	20.8	0.8	29.2	0.6
III	9.9	1.8	4.8	0.4	23.8	5.3	29.9	3.1
IV	8.3	1.4	5.6	0.7	22.4	2.2	31.2	2.1
V	5.4	0.2	11.6	0.1	17.8	0.9	29.2	2.5
VI	7.4	0.1	9.4	1.6	17.6	1.6	27.1	0.7
VII	7.7	1.3	6.7	0.8	21.3	1.5	37.2	0.6
VIII	5.7	1.0	5.8	0.7	26.3	1.9	32.6	1.6
Polyester	11.1	1.2	5.2	0.3	24.0	9.1	27.1	1.4
Polypropylene (70 g/m^2^)	10.0	1.2	5.6	0.3	24.5	5.1	28.4	1.9
Viscose	10.8	2.3	7.7	1.7	21.2	2.2	51.6	4.1

* Std dev—standard deviation.

**Table 4:** Results of thrombin assay as outlined in the Experimental Section.

Sample	nM thrombin	CV *	Velocity index	CV *
I	457.8	8.5	336.5	39.4
II	391.7	8.8	391.7	8.8
III	360.1	2.6	180.0	2.6
IV	442.8	0.9	442.8	0.9
V	373.7	8.0	186.8	8.0
VI	350.5	0.5	263.3	47.6
VII	360.1	0.4	360.1	0.4
VIII	385.9	8.3	158.9	20.2
Viscose	423.5	2.8	315.5	44.6
Polyester	461.9	3.2	N/A	–
Rayon/Polyester	418.3	1.5	418.3	1.5
Plasma Control	327.4	1.9	163.7	1.9
Control High/Buffer	288.2	3.5	N/A	–
Control Low/Buffer	187.2	1.0	77.9	27.3

* CV—coefficient of variation.

As seen in Table 2 an increase in hydropbhobicity and swell ratio of V–VIII corresponds to reduced time to fibrin formation (Table 3). Previously it was shown that the charge of polysaccharide-based fibers may be tunable to procoagulant properties [34]. This study further demonstrates how charge and the fiber surface area due to swelling may be designed to influence clotting as illustrated in Figure 3a. Most of the greige cotton/synthetic blends with the exception of IV elicited a reduction in clotting time, that correlates to a decrease in ζ_plateau_ with *K* (*K* = time from end of *R* to formation of 20 mm clot) as seen for samples V–VIII. The more hydrophilic material V has a ζ_plateau_ of –22 mV and the corresponding TEG-determined k value is 11.6 ± 0.1 min. Whereas, the hydrophobic VIII has a ζ_plateau_ of −57 mV and a corresponding k of 5.8 ± 0.7 min. As can be seen from the results of the 100% synthetic control fibers (XI, X, XI) including polyester, polypropylene, and viscose the R value is not reduced significantly below the blood control compared with the blended fibers. Thus the compositional nature of the synthetic fibers does appear to play a role in the contact activation coagulation component of the blends.

It is important to note that TEG does not assess properties of traumatic wound clotting that may be dependent on the gross dressing structure including dressing adherence, sealant properties related to rate of blood flow, rate of platelet uptake, and properties that influence interactions with the blood vessel endothelium.

### 2.3. Coagulation Cascade Contact Coagulation Properties 

The negative ζ_plateau_ and range in polarity of the materials prompts consideration of the mechanism of contact activation coagulation associated with the material. Contact activation coagulation or intrinsic coagulation is initiated by factor XII activation on negatively charged surfaces [35]. Two other plasma proteins, plasma prekallikrein, and high molecular weight kininogen (HMWK), participate in binding on the surface and activation of factor XII which triggers fibrin formation via its substrate factor XI.

Negatively charged surfaces present in kaolin and glass have long been known to initiate contact activation for over 50 years [36,37]. The term “glass effect” has traditionally described the observation that blood clots faster with polar surfaces than with nonpolar surfaces. Both negatively and positively charged surfaces have been known to activate different serine protease coagulation factors. For example negatively charged polyphosphate species are capable of enhancing fibrin clot structure via Factor XII [38], and positively charged materials have the ability to activate FVII [39]. It has also been shown that amine polymers like poly-lysine are capable of enhancing the activation of FVII, FX and FII [40]. Thus, there has been considerable research on the effects of charged surfaces on coagulation. It is interesting that the prothrombin time (PT) and activated partial thrombin time (aPTT) assay used to detect blood abnormalities were improved upon by adding a charged contact activator like kaolin, celite or ellagic acid [41]. In the presence of a contact activator the serine proteases of the intrinsic pathway are activated in descending order of Factor XIIa-Xia-IXa-Xa [42]. 

The significant differences in *R* values (time to fibrin formation) observed in this study for the different blends suggest that variations in contact activation may be mediated by surface charge and polarity differences among the samples examined. A variety of negatively charged surfaces are known to initiate FXII activation [35,43]. Historically, it has been proposed that FXII binds to negatively charged surfaces through positively charged amino acids in its heavy chain, or through complexation with HMWK that binds to surfaces in a similar manner [42,44]. More recently it has been illustrated that adsorption of FXII onto anionic surfaces may not be possible due to an “adsorption-dilution” of competing proteins from blood which dilute the putative adsorption of FXII and its protein complexes, and in effect create more protein determined adsorption [45]. Thus the mechanism of contact activation accounting for both competing protein adsorption and anionic hydrophilic and hydrophobic surfaces is complex. The relation of surface polarity (as measured by water contact angles) to contact activation has also recently been characterized for hydrophilic charged, hydrophobic and mixed monolayer surfaces. It was found by Sperling *et al.* [46] that surfaces that were mixed or intermediate between hydrophilic charged and hydrophobic had significantly higher thrombin levels associated with contact activation. In this regard it has been noted that surfaces having a mix of charged and hydrophobic groups promote a positive interaction between contact activation and platelet adhesion [46,47]. In this study an increase in negative charge and hydrophobicity are complementary and mediate material swelling that increases the rate of clotting as well. However, the surfaces of the materials of this study are more heterogeneous and considerably rougher than the controlled hydrophobic and charged surfaces reported by Sperling *et al.* [46]. The rougher surface of the nonwoven samples of this study also probably plays a role as well in clotting activity. Surface roughness in the intravasculature is associated with thrombogenicity [48]. As seen with the high contact angles hydrophobicity certainly plays an important role, and hydrophobicity and negative charge are classical switches for turning on contact activation. Thus, it appears that greige cotton can be used synergistically with more hydrophilic fibers, as seen here with viscose, to promote classic contact activation effects while introducing some wettability and absorption through the use of hydrophilic fiber that are essential to dressing development. The potential to use other more hydrophobic fibers to tune the clotting rate is also shown here.

Although the role of hydrophilic viscose, in the clotting function is not well understood it has previously been suggested [6] that multifiber hydrophilic/hydrophobic compositions experience initial protein adsorption and formation of a Vroman layer [49] with subsequent platelet interaction. This property is consistent as well in the TEG results of rayon/polyester and nonwoven blend V (greige cotton/viscose) as seen in Table 3, where a low *R* value is observed for a viscose-containing blend. Sample VI is identical in composition to V, but higher in material density. VI has a higher *R* value and is somewhat more hydrophilic and V is more hudrophobic giving a reduced *R* value which is consistent with the other structure/function findings of the paper and is reflected in the electrokinetic parameters (ζ_plateau_).

## 3. Experimental Section

A commercially available bale of pre-cleaned greige cotton (True Cotton™) [50] was acquired from T. J. Beall, LLC. A test matrix consisting of various types of hydroentangled nonwovens containing mechanically cleaned greige cotton fiber were produced in trials with Trützschler Nonwovens at their pilot facility in Wolfsgartenstraße 6, 63329 Egelsbach, Germany.

### 3.1. Thromboelastography

Thromboelastography was performed at 37 °C on a TEG 5000 Hemostasis System consisting of a Haemoscope Thrombelastograph Analyzer and TEG analytical software 4.2.3 (Haemoscope Corporation, Niles, IL, USA). Analysis was carried out by adding 90 μL calcium chloride (0.2 M) to 990 μL citrated bovine blood, and immediately adding 240 μL of the activated blood to the thromboelastography cups that contained 1 mg fabric sample in 20 μL citrated saline (5.375 mM disodium citrate, 146 mM NaCl). Multiple runs were performed on each sample.

### 3.2. Thrombin-Generation Assay

Thrombin generation was assayed using a modified fluorogenic method, Technothrombin TGA (Technoclone, Vienna, Austria). Approximately 10 mg fabric was added to the sample well of a 24-well microplate, with 200 μL TGA buffer (hepes-NaCl-buffer containing 0.5% bovine serum albumin). To this were added 300 μL TGA reagent D (phospholipid micelles in Tris-Hepes-NaCl buffer), 300 μL citrated bovine plasma (Quad Five, Ryegate, MT, USA) and 500 μL of the calcium-fluorogenic substrate (1 mM substrate; 15 mM CaCl_2_). Fluorescence readings were started immediately after addition of the substrate and were read for 60 minutes in 1 minute measurement intervals. Readings were made on a BioTek Synergy HT reader (BioTek Instruments, Winooski, VT, USA) at 37 °C.

### 3.3. Zeta Potential Measurements

The determination of the ζ-potential was carried out with the Electro Kinetic Analyzer (Anton Paar, Ashland, VA, USA) using the cylindrical cell developed for the measurement of fibrous samples. When a fiber absorbs liquid and swells, the surface charges become farther separated and the absolute value of its ζ-potential decreases. Two kinds of measurements were made on each sample: (1) swell tests to measure the rate and extent of fiber swelling (at a given pH) and (2) a pH titration in which the swelling is measured as a function of pH. All ζ-potential measurements were made in a 1 mM KCl electrolyte. 

In the electrokinetic apparatus the streaming potential is measured and the ζ-potential determined from the Smoluchowski equation:

(1) $$\text{ζ}=\frac{\text{d}U}{\text{d}P}\frac{\text{ηκ}}{{\text{ε}}_{r}{\text{ε}}_{0}}$$

where *U* is the streaming potential, the potential generated when an electrolyte is forced to flow over a stationary charged surface, *P* the pressure, ε*_r_* and ε_0_ the dielectric constant and the vacuum permittivity, η the viscosity and κ is the conductivity of the measuring fluid. Surface conductivity of the fibrous samples was not taken into account. 

pH titrations were performed over a pH range of 1.8 to 11 to ensure recording both the isoelectric point (IEP) and the plateau potential. (The IEP is the pH at which ζ = 0 and provides insights into the surface association/dissociation processes.) 

Swell tests and pH titrations were carried out on hydroentangled nonwoven fabrics cotton-polyester blends, cotton-nylon blends or cotton and cotton by-products. Swell tests were performed during which the zeta potential, ζ, was measured against time. These data were subsequently fit to a first-order decay equation

(2) $$-\frac{\text{dζ}}{\text{d}t}=k(\text{ζ}-{\text{ζ}}_{\infty })$$

where ζ_∞_ equals ζ at infinite time and *k* is the decay constant. Integrating

(3) $$\ln\left(\frac{\text{ζ}-{\text{ζ}}_{\infty }}{{\text{ζ}}_{\text{0}}-{\text{ζ}}_{\infty }}\right)=-kt$$

ζ_0_ is the integration constant and is ζ at time = 0, *i.e.*, the time when the fiber first contacts the electrolyte. A regression routine was developed to evaluate swell data for ζ_0_, ζ_∞_ and k. ζ_0_ and ζ_∞_ were then used to calculate a Δζ.

(4) $$\Delta \text{ζ}=\frac{{\text{ζ}}_{\text{0}}-{\text{ζ}}_{\infty }}{{\text{ζ}}_{\text{0}}}$$

A mathematical regression routine was written for MathCAD and used to determine three parameters in swell tests, ζ_0_, ζ_∞_, and *k*.

### 3.4. Time-Dependent/Swell Behavior

The swell behavior of the materials was measured using the Anton Paar analyzer with the cylindrical cell template. The sample was loaded into the cell, and rinsed with electrolyte solution. The experiment initiated when the electrolyte first contacted the sample. The flow rate was adjusted in the range of 60 to 100 mL/min by compression of the sample and the sample size. The initial pH of the sample varied between 5.5 and 6.0 to mimic the pH of urine. The pH was adjusted in this range with 0.1 M NaOH or HCl, as needed. In any given experiment, 30–35 data points were taken.

### 3.5. Environmental Scanning Electron Microscope

A Philips XL-30 Environmental Scanning Electron Microscope (ESEM) (FEI Company, Hillsboro, OR, USA) was used to image the specimens, operating at 10–13 kV. The samples were mounted on standard Cambridge 1/200 SEM stubs using double-stick photo adhesive tabs. They were coated with 60/40% gold/palladium in a Technics’ Hummer II sputter coater to a thickness of 20–30 nm.

### 3.6. Contact Angle Measurements

Contact angles were measured with a VCA Optima (AST Products, Billerica, MA, USA). 1 and 5 μL drops of distilled water were placed on various samples of griege cotton and blends and contact angles measured. The contact angle was determined by placing 5 “markers” on designated places on the edge of the drop and the software returns left- and right-side contact angles based on drop shape analysis. 12 replicate measurements were made on each sample. The resultant contact angles were averaged and the standard deviation about the mean calculated. These averages and standard deviations are presented, above.

## 4. Conclusions 

The results of this study demonstrate interesting trends between structure surface properties of greige cotton nonwoven blends, and their clotting times. For example the trend between increased swell ratio and decreased clotting time after fibrin formation is a result that has not to our knowledge been reported in the literature. It is evident from the results of this study that unbleached cotton has potential as an effective hemostatic material in nonwoven dressings. The significantly improved hemostatic activity of greige cotton nonwovens over rayon/polyester and viscose, which are used in a variety of commercially available hemostatic nonwoven dressings, is apparent. This study also highlights the relative contributions of hydrophilic *versus* hydrophobic fibers in clotting function on nonwoven fibers, and further demonstrates the importance of combining fibers of differing polarity to produce enhanced clotting functionality when fibrous materials are used in hemostatic dressings. Future studies will explore the significance of this clotting property to the corresponding material composition, dry surface area, and surface roughness.
